# Linking Nutrition, Maturation and Aging: From Thrifty Genes to the Spendthrift Phenotype

**DOI:** 10.18632/aging.100286

**Published:** 2011-02-20

**Authors:** David Stipp

**Affiliations:** Aging/Impact Journals LLC, Arlington, MA, USA

## INTRODUCTION

Nearly 50 years ago geneticist James Neel famously proposed that “thrifty genes” were important contributors to the rising prevalence of diabetes [1]. Such genes promote efficient use and conservation of food energy, he theorized, and thus were favored by natural selection to help our ancient ancestors cope with famines. Now widespread in various populations, they predispose to obesity and diabetes, abetting a tendency to prepare for famines that never come.

Though intuitively appealing, the theory has often been challenged. Perhaps the strongest objection has been that there's little evidence our ancestors faced frequent, high-mortality famines that would have selected for thrifty genotypes [2]. Recently, the theory's proponents have countered that thrifty genes' selective advantage probably had more to do with fertility than survival—women who rapidly deposited fat during periods of adequate nutrition would have been able to sustain relatively high reproductive rates during lean times and make larger contributions to the gene pool [3,4]. Here I propose an extension of this reproduction-centered version of Neel's theory that bears on aging. One of my key premises is that many windows of opportunity for reproductive booms occurred during the Holocene as agricultural innovations spread, periodically increasing food availability between times of nutritional stress. The periods of plenty selected for genotypes capable of rapidly ramping up fecundity as food intake increased. Sexually mature females would have quickly added fat—a certain level of maternal body fat is critical for reproduction [5]. Prepubertal females would have similarly added fat in conjunction with the acceleration of development and earlier onset of sexual maturity.

I believe the boom times' selection of genotypes prone to nutrition-cued accelerated development is having an especially problematic effect today because of widespread childhood overnutrition. Accelerated development, which enhanced reproductive success in the past, now has a pro-aging effect with rapidly growing costs. Indeed, when viewed through the lens of the antagonistic pleiotropy theory of aging [6], this effect seems anything but thrifty: It predisposes toward what might be called the spendthrift phenotype, characterized by chronic activation of pro-growth pathways—notably those involving mTOR, insulin, and insulin-like growth factor-1—that support rapid development and sexual maturation but that also underlie later senescence [7]. The modern fallout encompasses a much broader array of age-associated ills than the diabetes that prompted Neel's original hypothesis. Indeed, the spendthrift phenotype may well increase the age-associated risks of most if not all diseases of aging, like the ruinous adult legacy of flush, fast-living youth.

### The selective pressure exerted by famines

Neel's proposal that past famines reduced survival of individuals lacking thrifty genotypes has been invoked to explain, among other things, epidemic rates of type 2 diabetes that developed among various new-world populations after their adoption of western diets and lifestyles, such as the Central Pacific's Nauru Islanders [8,9]. However, it seems unlikely that Pacific island populations, dwelling on luxuriantly vegetated islands surrounded by fish-rich temperate waters, would have faced much risk of catastrophic famine [10]. Speakman argues that severe famines have generally been rare demographic events, occurring about once a century (mainly after the advent of agriculture-based societies), and that, in any case, they have usually posed a limited threat to viability, causing excess annual mortality of perhaps 5%, much of which has affected old, post-reproductive adults whose differential mortality plays no role in natural selection [2].

Fixing this weak link in Neel's original argument, thrifty-gene proponents, notably Prentice and colleagues, have emphasized the high selective intensity engendered by differences in fertility [4]. Nutritional stress potently suppresses fertility, they observe, and even seasonal food shortages in the developing world reduce conceptions by 30% to 50%. Although catastrophic, high-mortality famines may have been rare, there's considerable evidence that milder ones have frequently occurred during the Holocene [3,11]. The agricultural revolution that began about 12,000 years ago augmented the risk of famines as growing sedentary populations increasingly relied on a limited number of food sources vulnerable to droughts, diseases, and other threats. Shortages would have favored genotypes less vulnerable to curtailing of reproduction by nutritional stress. (Fertility is suppressed in women when fat stores fall below about 22% of body weight [5].)

Moreover, a pan-human tendency to store energy as fat to enhance reproduction probably existed long before the Holocene. As Wells notes, humans and their *Homo* ancestors are, in essence, “colonizing apes” whose global spread was likely aided by such a tendency [12]. Ample energy reserves would have enabled uninterrupted fertility during colonization of nutrition-ally parlous territories. Big brains make large energy demands, and meeting that demand is especially critical during early development. (Newborns' brains may account for 80% of basal energy metabolism [13].) *Homo* females' energy thrift, including the deposition of larger fat stores than males, was probably critical for sustained fertility and nursing of big-brained infants in marginal environments.

It should be noted that nutritional stress needn't select exclusively for adipogenic thrift. Other forms of metabolic and behavioral plasticity, such as a tendency to hoard food or to increase foraging avidity, could buffer vicissitudes in food supply [12]. (The induction of “hard foraging” behavior in rodents by calorie restriction may represent a case of such plasticity [14].) The ability of offspring to match their levels of energy demand to maternal energy supplies might facilitate the evolving of diverse strategies to cope with energy stress [12]. Such diversity may underlie the variability in adiposity observed across modern populations.

### The selective pressure exerted by agricultural innovations

Thrifty-gene proponents have generally stressed the role of famines, as have authorities on nutrition's effect on reproduction and pubertal timing. In her groundbreaking studies on reproduction and nutrition, for example, Frisch identified famines as exerting the salient selective pressure [5,15,16]. As she explained, females who reproduced when undernourished left no viable offspring and may themselves have failed to survive, thus had few if any descendants. Her argument's plausibility increased with the discovery that leptin permits sexual maturation to proceed by conveying a signal that fat stores are adequate for reproduction [17-19].

But periods of plenty may also have been important in shaping the tie between nutrition and pubertal timing. Genotypes that predispose to accelerated development and onset of puberty in response to food intake above the minimum needed to permit reproduction—precursors of today's spendthrift phenotype—would have been particularly advantageous after the advent of agriculture. Prosperous periods may have lasted for many years in different regions as successive waves of agricultural innovation swept through (although they would have been punctuated by famines as local or regional carrying capacity was exceeded, droughts occurred, or other factors curtailed harvests) [11,20,21].

About 11,000 years ago, for example, three key cereals—emmer wheat, einkorn wheat and barley—were domesticated over the course of a few centuries in the region near Jericho and then disseminated through the Fertile Crescent region of Southwest Asia [20]. By 9,000 years BP, lentils, peas, chickpeas, bitter vetch, and flax had also been domesticated and spread through the region, as had animal domesticates, including sheep, goats, cattle and pigs. Later waves of domestication made date palms, olives, melons, and leeks, as well as donkeys and camels, available to the Fertile Crescent's growing agrarian communities [20].

Domestication of each species was a multi-stage process, potentially resulting in extended periods of gradually increasing agricultural productivity. Expanding trade networks would have facilitated waves of increasing productivity via dissemination of new domesticates (or better versions of existing ones), as well as of nutrition-enhancing innovations such as fish traps, millstones, and new irrigation techniques [11]. Thus, multi-year periods of intensifying agricultural productivity probably occurred in various regions around the world during our ancestors' long, erratic transition from foraging to farming. Indeed, it seems very unlikely that sedentary, agrarian communities with rapidly growing populations would have formed unless such periods had occurred. The rate of population growth during the early agricultural era was about four times greater than it was during the Paleolithic era, and the world population rose from an estimated 6 million to around 250 million between 10,000 years BP and 1,000 BP [11].

Cultural and behavioral factors may also have favored genotypes that predispose to accelerated sexual maturation during periods of plenty. In many populations, for instance, men prefer women with large hips and thighs, morphological traits correlated with relatively high body mass index [BMI], fertility and earlier menarche [22]. (It's likely that such plumpness rarely led to health-threatening obesity in the past.) An intriguing 1989 study of the Kipsigis people of Kenya's Rift Valley Province, a semi-pastoralist population little affected by modernization, showed that earlier-maturing women had higher reproductive success than later-maturing ones, apparently because early menarche enabled longer reproductive lifespans [23]. (Reproductive success was measured by calculating number of surviving offspring per year of married life.) Kipsigis men, who preferred to marry polygynously and purchased brides with “bridewealth” payments negotiated with their future wives' families, were found to pay more for wives who reached menarche early than for later-maturing ones. The association between larger brideweath and early menarche occurred independently of brides' family wealth, grooms' wealth, and differences in education between bride and groom, and thus it appeared to result from a recognition by Kipsigis men of the higher reproductive success of early-maturing women.

### The modern acceleration of sexual maturation

The average age of menarche in Europe fell from 16 to 17 in the early 1800s to roughly 13 by 1960 [15]. During the same period, the age at menarche in the U.S. fell from just under 15 to less than 13. These remarkable changes are widely thought to have resulted from improvements in nutrition and health brought on by the Industrial Revolution. Indeed, the decreasing age of menarche was accompanied by increasing height and weight of girls and boys during the same period, and when growth leveled off as optimal conditions were approached, the age of menarche also stabilized [15]. A similar pattern has been observed in developing countries, such as urban China after 1979, which marked the beginning of the nation's transformation to a thriving market economy in the wake of reforms instigated by Deng Xiaoping—Chinese urban girls' age of menarche has fallen by 1.23 years at the same time that they have been getting taller and heavier [24].

The rising prevalence of obesity among American children over the past several decades has been accompanied by a further decrease in the age of menarche. Among U.S. 6- to 11-year-olds, the prevalence of overweight in white girls more than doubled to 11.6% between the 1960s and 2000, while the percentage of black girls in the same BMI range quadrupled to 22.2% [25]. During approximately the same period, the National Health Examination Survey [1963-1970] and NHANES III [1988-1994] showed that the average age at menarche dropped from 12.75 to 12.54 years [25].

Various theories have been proposed to explain the decreasing average age of menarche in recent decades, including the possible influence of environmental chemicals that disturb hormonal pathways regulating pubertal timing [26]. While the influence of such “endocrine disruptors” and other factors can't be ruled out, it appears that the broad secular trend can be adequately explained by the well-documented link between early nutrition and pubertal timing [25]. Among the evidence supporting this conclusion are longitudinal studies showing that high BMI in childhood is a significant risk factor for precocious puberty [27,28].

Does obesity cause early menarche, or does earlier menarche cause an estrogen-mediated tendency toward obesity? Investigators of the link between high BMI and early puberty have long debated this question. From an evolutionary perspective, however, there seems little point in the debate, because the selective pressures that favored genotypes predisposing to accelerated maturation in response to increased food intake would have also generally favored storing energy as fat to meet the demands of early reproduction and lactation by relatively young, small females. Consistent with this idea, recent genome-wide association studies have shown that a majority of the single nucleotide polymorphisms associated with high BMI to date are also at least nominally associated with early menarche [29].

In males the relationship between nutrition and pubertal timing is less clear than in females [25,30]. One reason is that there is no readily ascertained and easily recalled indicator of pubertal timing in boys, as there is in girls with the onset of menses. Evolutionary considerations suggest that the relationship between early nutrition, BMI and pace of development is likely to play out differently in boys and girls. Males' stored energy has little if any bearing on whether their offspring develop normally and are adequately nursed after birth, and thus volatility in food supplies wouldn't be expected to select for male genes that link food intake and stored fat to reproductive timing. Instead, a nutrition-triggered acceleration of the tempo of sexual maturation in boys might more likely be accompanied by relatively speedy increases in stature and strength, aspects of physical development supporting their earlier entry into post-pubertal competition for mates and resources. Several studies, though not all, are consistent with this expectation. Relatively high BMI in childhood, which in non-obese males is more closely tied to muscle mass than fat, was correlated with the earlier onset of puberty, height gain in childhood, and earlier reaching of peak height velocity in boys in a longitudinal Swedish-population study [31]. Other data show that boys with faster pubertal development tend to have been taller and heavier in early childhood, and to have had significantly greater muscle mass than later maturers at the same age [32,33]. Notably, girls undergo more rapid bone maturation than boys, a difference primarily driven by girls' higher estradiol levels, and early puberty in girls is associated with shorter adult height due to accelerated bone aging and early fusion of epiphyseal growth plates [34]. In contrast, early-maturing boys are taller at puberty, but their final adult stature isn't significantly associated with age at puberty [35].

In sum, there's considerable evidence that the tempo of development in girls has been on the rise for more than a century largely as a result of increasing food availability and, in recent decades, overnutrition. Limited evidence suggests a parallel pattern in boys. Both trends are consistent with past favoring of genotypes that predispose to accelerated development and sexual maturity in response to periodic windows of plenty.

### Mechanisms linking nutrition, sexual maturation and aging

The hypothesis presented here draws on an old idea: faster development goes with earlier aging and mortality. This notion, whose roots go back to Aristotle, inspired McCay's classic calorie restriction studies in the 1930s [36]. In recent years, it has informed research on the long-term effects of life trajectories set by early environmental cues [37]. A number of species have been shown to undergo accelerated compensatory development after retarded growth due to poor prior nutrition, but such acceleration often entails costs that aren't evident until late in adult life [38]. In modern human populations, overnutrition beginning early in life taps such plasticity to induce costs that never entered into the evolutionary calculus favoring genotypes predisposing to accelerated development. Life expectancy was less than 30 during most of human history, and thus few individuals lived long enough to show the downsides of such acceleration. Moreover, obesogenic lifestyles that induce the full-blown spendthrift phenotype, with rapidly rising prevalence of type 2 diabetes and other “diseases of aging” during adolescence, have been common only in recent decades [39].

Detailed mechanisms underlying “grow fast, die young” life trajectories are unclear, but various pieces of this puzzle are now in view. Multiple human studies have shown that the timing of puberty is closely linked to weight gain in infancy [40,41]. In addition, smaller size at birth is often followed by rapid catch-up weight gain before age 2, which in turn is correlated with the risk of obesity at 5 and 8 years of age, earlier puberty, and, in women, shorter adult stature [42,43]. These data make a strong case that a mechanism exists to accelerate development in response to food intake during early childhood.

Barker and colleagues have proposed that nutritional cues during gestation can exert profound effects [44,45]. In particular, they theorize that an undernourished pregnant woman communicates to her unborn baby that it is about to enter a food-short world, activating developmental programs that cause the baby to be born with a small body and metabolic traits geared to help it cope with nutritional stress—a “thrifty phenotype.” Later exposure to affluent lifestyles presumably increases such individuals' risk of coronary heart disease, type 2 diabetes and hypertension due to the mismatch between their thrifty phenotypes and post-infancy environments.

It's quite possible that such prenatal programming, which may involve epigenetic factors, can also be brought into play by maternal overnutrition, setting unborn offspring on trajectories of accelerated development. In keeping with this idea, mothers who undergo earlier menarche tend to have faster-growing babies that, by age 8, have larger body size than the offspring of later-menarche mothers [46]. As mentioned above, fast growth in infancy of such female offspring has been linked to increased odds that they too will undergo early menarche. Such transgenerational compounding of accelerated development might have helped maximize reproduction during past periods of plenty.

Major parts of the neurohormonal machinery underlying developmental plasticity, including pathways regulating the tempo of sexual maturity, have come to light in recent years. The discovery that leptin levels are correlated with the onset of puberty revealed a key conduit by which nutrition influences sexual maturation [18,19,47,48]. Leptin is secreted by adipocytes, and serum leptin levels, proportional to body fat, are thought to signal nutritional status to the hypothalamus and thus help govern appetite and energy expenditure. Animal and human data suggest that leptin permits puberty to proceed if fat stores are adequate [25]. However, leptin's role in sexual maturation may be more complex than this simple gating function. Leptin injections in prepubertal female rhesus monkeys cause earlier onset of menarche, and in humans leptin levels are inversely correlated with age of menarche, suggesting the hormone can help spur accelerated development in response to food intake [49,50].

Interestingly, transgenic “skinny” mice overexpressing leptin exhibit accelerated puberty despite having little if any adipose tissue, suggesting that leptin can have a pro-development effect irrespective of body fat [51]. After reaching sexual maturity early, the transgenic mice develop hypogonadism involving reduced activity of the hypothalamic-pituitary-gonadal axis, suggesting that leptin-mediated accelerated development can lead to early gonadal dysfunction and aging. A similar phenomenon may explain why precocious puberty in girls is a risk factor for adult polycystic ovarian syndrome, menstrual irregularities, and ovarian cancer [52]. In males, precocious puberty increases the risk of testicular and prostate cancer [52].

Early menarche is also linked to hyperinsulinemia, insulin resistance, and high insulin-like growth factor 1 [IGF-1]. levels. Insulin/IGF-1 signaling (IIS) has a pro-growth effect and thus may play a central role in accelerating development and reproduction [53]. When insulin-related pathways are chronically activated by overnutrition, however, compensatory feedback mechanisms lead to increasing insulin resistance, which is associated with diseases of aging [54]. Studies across diverse taxa have shown that attenuation of IIS signaling by calorie restriction and IIS-related mutations is closely tied to slow aging and extended lifespan, and to improved old-age survival [54,55]. One interpretation of these data is that nutrition-stimulated IIS early in life abets the spendthrift phenotype's program of accelerated development, and that chronic, post-maturational activation of IIS-related pathways is a key contributor to the phenotype's pro-aging effects.

Recently, genome-wide association studies have identified a number of loci correlated with pubertal timing and BMI. One of the first such studies linked an intronic polymorphism of the LIN28B gene to early menarche, and it appears that LIN28B helps control pubertal timing in both girls and boys, as well as the tempo of height development in both sexes [56-59]. The LIN28B variant linked to early menarche is also associated with long-term effects on growth and risk of obesity-correlated diseases [59]. LIN28B is an important regulator of microRNA activity involved in the timing of development—seminal studies in *Caenorhabditis elegans* have shown that worms' lin-28 gene, homologous to LIN28B, controls the rate of progression through larval stages to adult cuticle formation [60]. The LIN28 pathway has also been implicated in tumor growth, stem cell pluripotency and aging [61].

A recent meta-analysis of 32 genome-wide association studies identified 30 new loci correlated with age at menarche, including four previously linked to BMI [29]. In addition to the BMI-linked loci, three of the novel menarche loci were in or near genes thought to be involved in energy homeostasis, including CRTC1, which encodes the CREB-regulated transcription coactivator 1. Transgenic mice lacking functional CRTC1 genes are hyperphagic, obese, and, in one study, infertile. Earlier studies also suggested that leptin potentiates CRTC1's activity, which in turn is tied to increased secretion of gonadotropin-releasing hormone that triggers the onset of puberty [62,63].

Other research has suggested that mTOR [mammalian target of rapamycin], an important intracellular energy sensor and growth regulator, also helps regulate pubertal timing and reproductive function [63]. Inhibiting mTOR with rapamycin delays puberty onset in female rats, indicating that mTOR is a central component of a regulatory network in the brain that modulates fertility and sexual maturation based on energy status. This network is also thought to involve AMPK (AMP-activated protein kinase), another pivotal nutrition sensor known to interact with mTOR [63]. (AMPK, whose activity is enhanced by nutritional stress, deactivates mTOR.) Like mTOR inhibition, AMPK activation can delay puberty, and treatment with metformin, which activates AMPK, has been shown to normalize pubertal timing in low-birthweight girls at risk of early menarche [64].

Of special interest here is the fact that mTOR and AMPK are deeply implicated in aging. Studies in many species have shown that calorie restriction can extend lifespan, and this anti-aging effect appears to be critically dependent on suppression of the conserved TOR pathway, which integrates nutrition and growth-factor signals to regulate protein synthesis, cell growth and proliferation, autophagy, and other cellular functions [7,65-67]. Recent pioneering studies have shown that inhibiting mTOR with rapamycin extends mouse lifespan. Remarkably, this longevity enhancement has been observed when administration of the drug is initiated as late 20 months of age [68,69]. Rapamycin also prevented age-dependent obesity, slowed aging and delayed cancer in cancer-prone mice [70]. In contrast, chronic activation of the TOR pathway, which occurs in cases of overnutrition, is linked to accelerated aging and early mortality [66].

AMPK helps regulate energy balance, insulin signaling, fatty-acid oxidation, and mitochondrial biogenesis [7,71,72]. Aging-associated decline of AMPK activity is thought to contribute to reduced mitochondrial function, insulin resistance, and other correlates of aging [73]. Chronic activation of AMPK by administering metformin to HER-2/neu female mice and female outbred SHR mice extends mean and maximum lifespan, possibly in part via indirect inhibition of mTOR [74,75]. AMPK's important role in aging is further underscored by the evidence (Anisimov et al. in this issue of *Aging*) that metformin treatment is more effective in prolonging life span in female mice when started early in life [76].

These intersecting lines of evidence suggest that chronic activation of mTOR (concomitant with low AMPK activity) by early overnutrition plays a central role in eliciting the spendthrift phenotype. As Blagosklonny has observed, once a developmental program is switched on, it isn't necessarily switched off, even if its continuation is harmful later in life [7]. Elaborating on this insight, which follows from the antagonistic pleiotropy theory of aging, he argues that TOR's growth-promoting and other activities are critical for development, and that purposeless, post-developmental continuation of TOR activity, in conjunction with cell-cycle arrest (a conserved function necessary to prevent cancer), is a key driver of cellular “hyper-function” that leads to hypertrophy, hyperplasia and cell senescence underlying the aging process. The damaging effects of such hyper-function include insulin resistance and pro-inflammatory processes, pro-cancer mitogenic stimulation, osteoclast-driven bone loss, and the proliferation and hypertrophy of arterial smooth muscle cells.

I believe that the spendthrift phenotype represents an exaggerated version of Blagosklonny's “quasi-programmed aging,” and that an important source of this exaggeration is the collision of modern lifestyles with genotypes that were favored during past periods of plenty. To slightly amend an analogy used by Blagosklonny, the spendthrift phenotype is like a car without brakes whose gas pedal has been floored since it was put on the road.

### Implications

My hypothesis has several testable implications. For instance, it suggests that early-menarche loci associated with high BMI are underrepresented in centenarians.

The hypothesis also suggests that health risks associated with early overnutrition aren't simply consequences of obesity. As noted above, increased adiposity is one of the spendthrift phenotype's co-selected traits, and to some extent the obesity associated with the phenotype is secondary to, and potentiated by, activation of mTOR, IIS and other pro-growth pathways enlisted to accelerate growth, sexual maturation and reproduction. The pro-aging effect of chronically activating these pathways from an early age probably increases the age-associated risk of many adult diseases in tandem with overweight and obesity, including ones whose tie with adiposity has only recently become apparent, such as dementia [77]. Consistent with this idea, women who experience early menarche are at heightened risk of all-cause mortality, and a recent study suggested this across-the-board risk is only partly mediated by increased adiposity [78].

If the hypothesis is correct, efforts to address childhood obesity would most effectively lower future health risks if they include focused efforts to interrupt early metabolic programming that elicits the spendthrift phenotype. For example, preventing rapid weight gain in infancy, as well as maternal overnutrition during pregnancy, may suppress the program during a critical window of time in early life, lessening its pro-aging effects more effectively than later interventions. Similarly, metformin's puberty-delaying effect suggests that it may be particularly effective at reducing the phenotype's adult health risks—its wider use in pediatric medicine probably makes sense. Better understanding of genes and pathways underlying the phenotype's pattern of fast development should suggest other drug targets that could yield highly effective preventive medicines.
